# Relationship between Vision-Related Quality of Life and Central 10° of the Binocular Integrated Visual Field in Advanced Glaucoma

**DOI:** 10.1038/s41598-019-50677-0

**Published:** 2019-10-18

**Authors:** Yoshio Yamazaki, Kenji Sugisaki, Makoto Araie, Hiroshi Murata, Akiyasu Kanamori, Toshihiro Inoue, Shinichiro Ishikawa, Keiji Yoshikawa, Hidetaka Maeda, Yuko Yamada, Akira Negi, Masaru Inatani, Hidenobu Tanihara, Satoshi Okinami, Kenji Mizuki, Koichi Mishima, Kenichi Uchida, Shun Matsumoto

**Affiliations:** 1 Department of Ophthalmology, Tokai University Tokyo Hospital, Tokyo, Japan; 2 Department of Ophthalmology, International University of Health and Welfare, Mita Hospital, Tokyo, Japan; 3 Department of Ophthalmology, University of Tokyo Graduate School of Medicine, Tokyo, Japan; 4 Department of Ophthalmology, Kanto Central Hospital of the Mutual Aid Association of Public School Teachers, Tokyo, Japan; 5 Department of Surgery, Division of Ophthalmology, Kobe University Graduate School of Medicine, Kobe, Japan; 6 Department of Ophthalmology, University of Kumamoto Graduate School of Medicine, Kumamoto, Japan; 7 Department of Ophthalmology, University of Saga Graduate School of Medicine, Saga, Japan; 8 Yoshikawa Eye Clinic, Machida, Japan; 9 Department of Ophthalmology, Nihon University School of Medicine, Tokyo, Japan; 10 Department of Ophthalmology, Tokyo Post and Telecommunication Hospital, Tokyo, Japan

**Keywords:** Glaucoma, Quality of life

## Abstract

To investigate the relationships between sensitivity loss in various subfields of the central 10° of the binocular integrated visual field (IVF) and vision-related quality of life (VRQoL) in 172 patients with advanced glaucoma. Using the Random Forest algorithm, which controls for inter-correlations among various subfields of the IVF, we analysed the relationships among the Rasch analysis-derived person ability index (RADPAI), age, best-corrected visual acuity (BCVA), mean total deviations (mTDs) of eight quadrant subfields in the IVF measured with the Humphrey Field Analyzer (HFA) 10-2 program (10-2 IVF), and mTDs of the upper/lower hemifields in the IVF measured with the HFA 24-2 program (24-2 IVF). Significant contributors to RADPAIs were as follows: the inner and outer lower-right quadrants of the 10-2 IVF contributed to the dining and total tasks; the lower-left quadrant of the 10-2 IVF contributed to the walking, going out and total tasks; the lower hemifield of the 24-2 IVF contributed to the walking, going out, dining, miscellaneous and total tasks; and BCVA contributed more to the letter, sentence, dressing and miscellaneous tasks than to others. The impact of damage in different 10-2 IVF subfields differed significantly across daily tasks in patients with advanced glaucoma.

## Introduction

Glaucoma causes progressive, irreversible optic nerve damage and visual field (VF) damage^1^; thus, it consistently ranks among the leading causes of blindness, accounting for 25% of all cases of blindness in Japan^2^. Blindness greatly impacts quality of life; therefore, a primary goal of glaucoma treatment is to preserve the patient’s vision-related quality of life (VRQoL). In the context of providing appropriate public care and support, the associations between VF damage and VRQoL in glaucoma patients need to be more fully understood.

In 1967, using a conventional tangent screen, Esterman^3–5^ presented a grid for evaluating VFs quantitatively, from the centre to the periphery^3^. In that same study, Esterman proposed a scoring system for peripheral acuity, consisting of 100 grids based on the American Medical Association standard isopter, and derived the Esterman disability score^4^. The original Esterman disability score assessed the percentage of points seen, using suprathreshold stimuli, in monocular fields. In 1982, to better approximate real-life patient experience, a method for functional scoring of binocular VFs was developed, which has been used for individual and mass screenings as a standard for all patients, on all existing manual or computerized perimeters^5^. Mills and Drance used a computerized perimeter to examine the Esterman disability score for binocular VF in patients with advanced glaucoma, revealing that Esterman disability scores correlate with patients’ perceptions of their visual disabilities^6^.

After the popularization of computerized automated perimeters, central field thresholds became important for detecting early glaucomatous changes. Crabb *et al*.^7^ proposed an integrated binocular VF score that was based on simultaneous binocular VF results, using the maximum sensitivity from each of corresponding points; they found that integrated VF (IVF) scores were more strongly associated with self-reported visual disabilities than were Esterman disability scores. To predict binocular IVF sensitivity at each test point, Nelson-Quigg *et al*.^8^ proposed a model based on the combination of the higher sensitivity between the two eyes at each VF location—the “best location” model—and the binocular summation model, calculated as the binocular summation of sensitivity between the eyes at each location. Jampel *et al*.^9^ reported findings to similar those of Crabb *et al*.^7^.

Sumi *et al*. developed a questionnaire specialized for the assessing VRQoL of Japanese glaucoma patients^10,11^ (Appendix). The questionnaire was used to examine the relationship between VRQoL and mean retinal sensitivity for a cluster in the binocular IVF, using a Humphrey Field Analyzer (HFA; Carl Zeiss, Dublin, CA, USA) with the Swedish Interactive Threshold Algorithm standard (SITA-S) central 30-2 program (HFA 30-2) that followed the best location model. They evaluated the impact of damage in different subfields on the performance of different daily tasks and demonstrated that sensitivity in the lower central 5° of the VF was most important for the VRQoL of glaucoma patients^10^. In patients with glaucoma, VF defects close to the point of fixation are more likely to threaten central vision than are defects farther into the periphery^12^; notably, the central VF, which is very important for visual function, is usually preserved until the end stages of glaucoma^6,12–16^.

The HFA SITA-S central 10-2 program (HFA 10-2) provides much more detailed information regarding visual function in the central VF, than do the HFA SITA-S central 24-2 (HFA 24-2) or 30-2 programs. The HFA 10-2 is a standard test for glaucoma patients that measures sensitivity at 68 test points, 2° apart from each other, in the central 10° of the VF. Despite the clinical importance of the central 10° of the VF in glaucoma patients, especially in cases with advanced damage^5,10,16–19^, no previous studies have investigated the relationship between HFA 10-2 test results and VRQoL in glaucoma patients.

Therefore, the purpose of this study was to further clarify associations between VRQoL and regions of sensitivity loss in the central 10° of the binocular IVF, in patients with advanced glaucoma, using HFA 10-2 test-program results.

## Results

**Figure 1:**
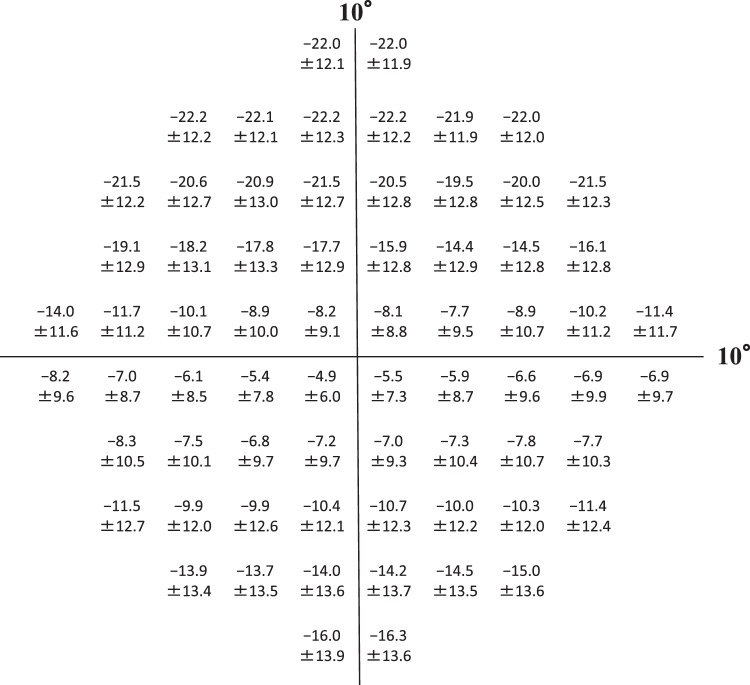
Mean total deviations of each test point, as determined using the binocular integrated visual field of the Humphrey Field Analyzer Swedish Interactive Threshold Algorithm standard 10-2 (10-2 IVF). Values are presented as mean total deviation ± standard deviation, in decibels.

**Figure 2:**
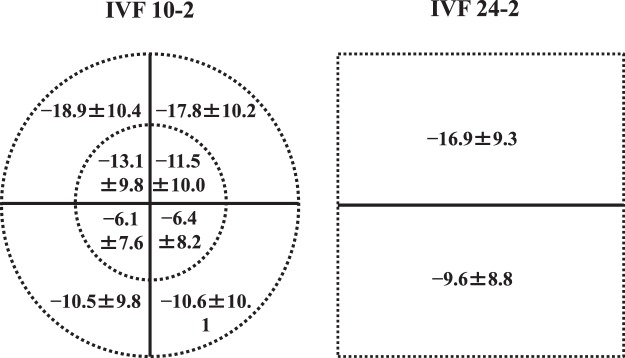
Mean total deviations (mTDs) of subfields, as determined using the binocular integrated visual field (IVF) of the Humphrey Field Analyzer (HFA) Swedish Interactive Threshold Algorithm standard (SITA). Left: mTDs of eight quadrant subfields within 5° of the fixation point, as determined using the binocular IVF of HFA SITA 10-2. Right: mTDs of upper/lower hemifields as determined using the binocular IVF of HFA SITA 24-2. Values are presented as mean total deviation ± standard deviation (decibels) for each subfield.

**Figure 3:**
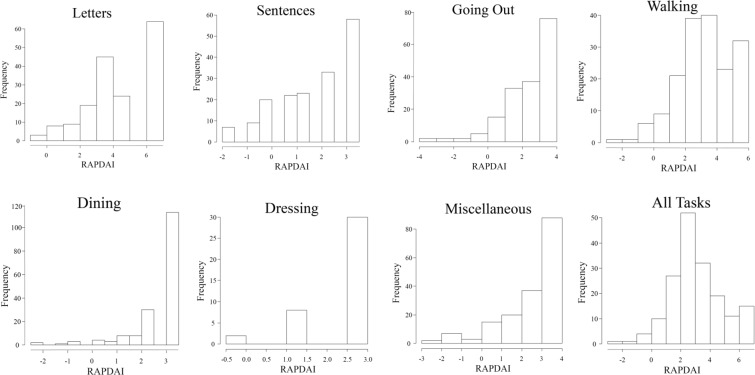
Histograms of the relationships between the Rasch analysis-derived personal ability index (RADPAI) for each of seven tasks and for all tasks, based on the frequency of subject responses. The greater the RADPAI, the better the patient’s vision-related quality of life is.

We evaluated VRQoL in 172 patients (55 males, 117 females) with advanced glaucoma; 138 patients had primary open-angle glaucoma, 8 had primary angle-closure glaucoma, 6 had pseudoexfoliation glaucoma, 4 had developmental glaucoma and 16 had secondary glaucoma. Patient characteristics are summarized in Table 1. The mean total deviations (mTDs) for each tested point, each of the eight quadrant subfields of a binocular integrated HFA 10-2 VF (10-2 IVF) and the upper/lower hemifields of a binocular integrated HFA 24-2 VF (24-2 IVF) are shown in Figs 1, 2. In comparing mTDs among the eight subfields of the 10-2 IVF, we found significant differences [analysis of variance, p < 0.0001]. The mTDs of the right and left inner lower quadrant subfields showed significantly less damage than those of the other subfields (Scheffe^20^, p < 0.001). The mTDs of the upper hemifield of the 24-2 IVF showed significantly more damage than those of the lower hemifield (paired t-test, p < 0.001) (Fig. 2). The histograms in Fig. 3 show the frequency of occurrence of the different Rasch analysis-derived personal ability index (RADPAI) values, across subjects, for each of the seven tasks and for all tasks combined.

**Table 1:** Patient demographics for enrolled patients with advanced glaucoma (n = 172).

Age	62.5 ± 12.2^a^
Gender (male: female)	117: 55
Refraction of better eye (diopter)	−2.9 ± 4.3
Refraction of worse eye (diopter)	−2.7 ± 3.9
IOP^b^ of better eye (mmHg)	12.8 ± 2.9
IOP of worse eye (mmHg)	12.9 ± 2.6
MD^c^ of better eye (dB^h^)	−17.0 ± 8.2
MD of worse eye (dB)	−26.3 ± 3.0
BCVA^d^ in better eye (log MAR^e^)	−0.04 ± 0.14
BCVA in worse eye (log MAR)	0.21 ± 0.46
mean TD^f^ of 24-2 IVF^g^ (dB)	−15.2 ± 7.4
mean TD of 10-2 IVF (dB)	−13.2 ± 7.7

^a^Values are expressed as mean ± standard deviation.

^b^IOP, intraocular pressure.

^c^MD, mean deviation.

^d^BCVA, best-corrected visual acuity.

^e^Log MAR, logarithm of minimum angle of resolution.

^f^TD, total deviation.

^g^IVF, integrated visual field.

^h^dB, decibel.

Table 2 shows the Spearman correlations between ages, the best-corrected visual acuity (BCVA) in the logarithm of the minimum angle of resolution (log MAR) in the better-VA eye (VA in the better eye) and worse eye (VA in the worse eye), mTDs of the eight quadrant subfields of the 10-2 IVFs and of the upper/lower hemifields of the 24-2 IVFs, and the RADPAI. RADPAI values indicate the relative difficulty, from Rasch analysis^11^, for each of the seven tasks and for all tasks combined, as determined using Sumi’s VRQoL questionnaire (Appendix). In general, the VA in both eyes, the mTDs of the paracentral lower quadrant subfields of the 10-2 IVF and of the lower hemifield of the 24-2 IVF were significantly correlated variables with all VRQoL tasks, except for dressing. None of the VRQoL tasks were significantly correlated with the mTDs of the upper left quadrant subfields after Bonferroni’s correction (P > 0.05/104 = 0.00048).

**Table 2:** Correlations among RADPAI, Age, Visual Acuity and Visual Field Measurements.

RADPAI	Letters	Sentences	Walking	Going Out	Dining	Dressing	Miscellaneous	For all tasks
Age	NS	NS	NS	NS	NS	NS	NS	NS
BCVA in better eye^a^	−0.385	−0.403	−0.269	−0.274	−0.246	NS	−0.377	−0.349
BCVA in worse eye^b^	NS	−0.390	−0.259	−0.282	−0.300	NS	−0.307	−0.304
**10-2 IVF (quadrant subfield)**								
Outer Upper right	NS	NS	0.322	NS	NS	NS	0.256	0.306
Inner Upper right	0.303	0.298	0.307	NS	NS	NS	NS	0.311
Outer Upper left	NS	NS	NS	NS	NS	NS	NS	NS
Inner Upper left	NS	NS	NS	NS	NS	NS	NS	NS
Outer Lower left	0.331	0.357	0.360	0.310	0.359	NS	0.401	0.414
Inner Lower left	0.353	0.381	0.356	0.352	0.358	NS	0.361	0.415
Outer Lower right	0.340	0.397	0.394	0.334	0.415	NS	0.387	0.440
Inner Lower right	0.353	0.460	0.372	0.389	0.464	NS	0.396	0.449
**24-2 IVF (hemifield)**								
Upper	NS	NS	0.299	NS	NS	NS	NS	0.265
Lower	0.336	0.346	0.425	0.322	0.371	NS	0.383	0.458

Spearman correlation coefficients between Rasch analysis-derived personal disability index (RADPAI) values, and age and visual acuities (VAs) and mean Total Deviations (TDs) of subfields in the binocular integrated visual field (IVFs) from 10-2 and 24-2.

^a^BCVA in better eye, best-corrected visual acuity in log MAR in better-VA eye.

^b^BCVA in worse eye, best-corrected visual acuity in log MAR in worse-VA eye.

NS indicates no significant correlation after Bonferroni’s correction (P > 0.05/104 = 0.00048).

Table 3 shows the statistical significance of related variables for each VRQoL task and overall VRQoL, as determined using the Random Forest method^21^. VA in the better eye significantly contributed to higher performance ratings in patient-reported ability regarding letters, sentences, dressing and miscellaneous activities. For the letters and dressing tasks, VA in the better eye was the only significant factor contributing to patient-reported difficulty; patient-reported difficulty with the letters and dressing tasks was not significantly influenced by the mTDs of the 10-2 IVF quadrant subfields or those of the upper/lower hemifields of the 24-2 IVF. The contribution of 10-2 IVF quadrant subfields varied according to the task. Age did not significantly influence patients’ VRQoL. No parameters which did failed to show significant Spearman correlation coefficients to VRQoL tasks (Table 3) were selected as significantly contributing factors by the Random Forest method^21^.

**Table 3:** Rank importance of related variables for each vision-related quality of life (VRQoL) task.

Rank	Letters	Sentences	Walking	Going Out	Dining	Dressing	Miscellaneous	Total Disability Index
1	BCVA in better eye^a^ (0.0020)	IVF 10-2^c^ Inner Lower right (0.0010)	IVF 10-2 Inner Lower left (0.0005)	IVF 10-2 Inner Lower left (0.0010)	IVF 24-2 Lower (0.0015)	BCVA in better eye (0.0425)	IVF 10-2^c^ Outer Lower left (0.0105)	IVF 24-2 Lower (0.0001)
2		IVF 24-2^d^ Lower (0.0065)	IVF 24-2 Lower (0.0075)	IVF 24-2 Lower (0.0285)	IVF 10-2 Outer Lower right (0.0020)		BCVA in better eye (0.0145)	IVF 10-2 Inner Lower left (0.0010)
3		IVF 10-2 Outer Lower right (0.0070)		IVF 10-2 Inner Lower right (0.0305)	IVF 10-2 Inner Lower right (0.0230)		IVF 10-2 Inner Lower right (0.0290)	IVF 10-2 Inner Lower right (0.0225)
4		BCVA in worse eye^b^ (0.0095)					IVF 24-2 Lower (0.0350)	
5		BCVA in better eye (0.0265)						

Rank importance of related variables was identified according to significance levels, as calculated with the Random Forest machine-learning algorithm.

VRQoL, vision-related quality of life.

^a^BCVA in better eye, best-corrected visual acuity in log MAR in better-VA eye.

^b^BCVA in worse eye, best-corrected visual acuity in log MAR in worse-VA eye.

^c^IVF 10-2, binocular integrated visual field (IVF) of Humphrey central 10-2 test program.

^d^IVF 24-2, binocular integrated visual field (IVF) of Humphrey central 24-2 test program.

Outer (inner) lower right (left) indicates outer (inner) lower right (left) quadrant subfield of binocular integrated visual field of Humphrey central 10-2 test program.

The values in parentheses are significance levels calculated with the Random Forest machine-learning algorithm.

**Figure 4:**
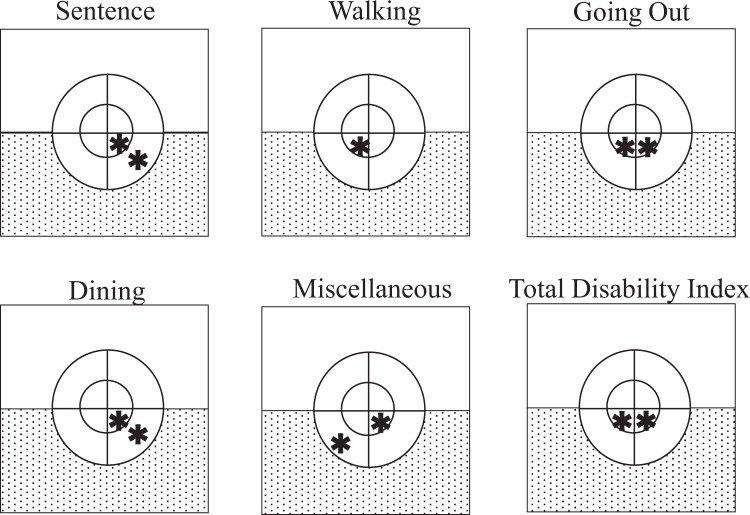
The location of the subfield(s) that significantly contributed to the vision-related quality of life for each task. Shown are the eight quadrant subfields of the binocular integrated visual field (IVF) of Humphrey Field Analyzer Swedish Interactive Threshold Algorithm standard (SITA) 10-2. The two square fields indicate the upper and lower hemifields of 24-2. Each * indicates a significantly contributing subfield, as determined using the Random Forest machine-learning algorithm (p < 0.05). The sand pattern square of the lower hemifield of 24-2 also significantly contributed (p < 0.05).

Figure 4 summarizes the 10-2 and 24-2 IVF subfields that contributed significantly to VRQoL for each task. The inner and outer lower right quadrant subfields of the 10-2 IVF contributed to the sentence task; the inner lower left quadrant subfield of the 10-2 IVF contributed to the combined tasks of walking and going out; the inner lower right quadrant subfield of the 10-2 IVF also contributed to going out. Finally, the outer and inner lower right quadrant subfields of the 10-2 IVF contributed to the dining task. The lower hemifield of the 24-2 IVF contributed to all tasks except letters and dressing. In brief, the entire 5° inferior subfield and the outer lower right quadrant subfield of the 10-2 IVF, as well as the lower hemifield of the 24-2 IVF, were found to be most important for VRQoL.

## Discussion

To our knowledge, this is the first study reporting the relationship between visual disability and subfields in the central 10° of the binocular IVF, as constructed from HFA 10-2 test results, in patients with advanced glaucomatous VF damage.

The central part of the VF is functionally more important than the peripheral parts, and the lower central VF may be the most important region^3,10,16,18,22^. The most commonly used automated perimetry programs for glaucoma are the HFA 24-2 and HFA 30-2 programs, which use 54 or 76 test points that are 6° apart, and which only include 12 test points located within 10° of the fixation point. In contrast, the HFA 10-2 test program closely assesses the central 10° of the VF, using 68 test points that are 2° apart. Glaucomatous VF defects are reportedly detected more readily when a VF test strategy is used that includes more closely spaced test points^23,24^. Therefore, the HFA 10-2 test program should provide more detailed and important spatial information in the central region of VF, relative to the HFA 24-2 and HFA 30-2 test programs.

As summarized in Table 2, damage within different 10-2 IVF subfields showed similar Spearman correlation coefficients for the same VRQoL tasks, and significant inter-correlations across 10-2 IVF subfields are to be expected. Thus, to evaluate the relationships between VRQoL-related parameters and BCVA, as well as between VRQoL-related parameters and VF damage in various subfields, it is important to control for such inter-correlations. The advantage of the Random Forest algorithm^21^, used in the current study, is that it can control for inter-correlation of multiple explanatory variables; thus, it is particularly useful in analysing data that include test results obtained from various subfields of the VF^17,25,26^.

In the current study, for VRQoL in general, the contribution of the lower right and/or left inner quadrant subfields in the central 10-2 IVF was more evident than were the contributions from the other subfields. That is consistent with earlier findings that losses of retinal sensitivity in the lower central hemifield were more related to visual disability in daily life than were losses of retinal sensitivity in the upper central hemifield^10^. Furthermore, the current study demonstrated apparent differences in the use of crucial central 10° subfields for different types of daily tasks.

Our study suggests that, for the sentence task, the inner and outer right quadrant subfields of the 10-2 IVF are more important than are the left subfields and BCVA in both eyes. It seems logical that a sentence task requires a wider field of perception than does a letter task, where only VA in the better eye, that is, good central vision, is important. Japanese newspapers and books are usually read vertically from the upper right, whereas English newspapers and books are read horizontally from left to right. The left cerebral hemisphere is more crucial for reading than is the right hemisphere^27^, and the visual-perceptual mechanisms for word recognition are primarily localized to the left hemisphere^28^. It was reported that reaction times to light stimuli in the right VF were longer than those for stimuli in the left VF^29,30^, suggesting that given a similar extent of damage, damage in the right VF would cause more problems than that in the left VF. These results are compatible with the current finding suggesting that the inner and outer lower right quadrant subfields of the 10-2 IVF are more important for the sentences tasks, representing Japanese reading habits.

The current results also suggest that the inner and outer lower right quadrant subfields of 10-2 IVF and the lower hemifield of 24-2 IVF contribute more to dining tasks. According to Japanese custom, the main dishes are usually placed on the right side of the table. If a person with right subfield loss eats a meal, that person notices more inconvenience than a person with left subfield loss because of visual-perceptual asymmetry, as discussed previously^27–30^. The lower hemifield of the 24-2 IVF should also contribute to acknowledging the entire dining table.

The inner lower left quadrant subfield and the lower hemifield of the 24-2 IVF significantly contributed to the walking and going out tasks. In Japan, cars are driven on the left side of the road (pedestrians must walk along the right side of the road). It seems understandable, therefore, that a person who has left lower central subfield loss would likely feel inconvenienced and anxious while walking down the right side of the road. The inner lower right quadrant subfield of the 10-2 IVF should also contribute to going out. The lower hemifield of the 24-2 IVF contributed to miscellaneous tasks that include looking for objects dropped on the floor and other tasks requiring good central vision.

The present study suggests that VA in the better eye contributes more to the letters and dressing tasks than do the central visual subfields. This finding suggests that, for recognizing letters rather than sentences, and for buttoning and unbuttoning clothing, central vision would be more critical than a specific VF. It remains controversial whether VA in the better eye or in the worse eye has a stronger influence on visual disability in daily life. Several investigators^10,31,32^ have reported that VA in the better eye is the most important variable for VRQoL in glaucoma patients; however, Murata *et al*.^17^ reported that VA in the worse eye tended to have a greater impact on VRQoL than VA in the better eye. In previous studies, there have been no limitations on the VA of enrolled subjects^10,17,31,32^, and differences in VA in the two eyes might have been substantial in some study subjects. Importantly, all subjects in our study exhibited a decimal VA of ≥0.5 in each eye, so that precise binocular IVFs could be constructed. This difference might at least partly explain the discrepancy between our current results and the findings of Murata *et al*.^17^.

In Japan, official certification of visual impairment requires evaluation of the extent of VF impairment, as determined by Goldmann perimetry. The American Medical Association method for evaluation of VF impairment is based on the extent of the VF along each of the eight 45-degree meridians of the VF, as measured around eight meridians in the Goldmann perimeter^33^. Our results suggest that, in advanced stages of glaucoma, different subfields in the central 10° of the IVF contribute differently to VRQoL, depending on the type of task involved. This suggests that the central 10° of the IVF should be evaluated for official certification of visual impairment.

There are several limitations in the current study. First, because this study included only glaucoma patients with advanced VF damage, the current results regarding 10-2 IVFs are not necessarily applicable to all glaucoma patients, especially those patients with early VF damage. Second, Sumi’s questionnaire was developed for Japanese glaucoma patients who usually read vertically. Thus, results regarding sentence tasks would not be applicable to patients who usually read horizontally. Third, Sumi’s questionnaire does not include questions about driving or using electronic equipment, such as computers and smartphones, which should be evaluated in relation to VRQoL in modern life. Last, the rank contributions of 10-2 IVF subfields, for each VRQoL task in the current subjects, were obtained using a recently developed statistical method^21^, which may need further verification in future studies.

In summary, using Sumi’s VRQoL questionnaire and testing the central 10° of the binocular IVF in patients with advanced glaucoma, we found that damage in different central subfields of the VF significantly contributed to the success in performing different daily tasks that are important to quality of life. Evaluation of VA and binocular integrated HFA 30-2 or 24-2 VFs, along with subfield analyses of the central 10° of the binocular IVF, should provide a useful assessment and increased understanding of VRQoL in patients with advanced glaucoma.

## Methods

The current study was completed as part of the Advanced Glaucoma Study, a non-interventional, longitudinal, observational study performed by the Japan Glaucoma Society. The study protocol was approved by the ethics review committees of Nihon University School of Medicine, The University of Tokyo Graduate School of Medicine, Kobe University Graduate School of Medicine, Kumamoto University Graduate School of Medicine, Saga University Graduate School of Medicine and the Yoshikawa Eye Clinic of the Tokyo Post and Telecommunication Hospital. Written consent was given by patients for their information to be stored in the hospital database and used for research. This study was registered as protocol number UMIN000001004; this protocol adhered to the tenets of the Declaration of Helsinki.

### Subjects

Patients with advanced glaucomatous VF damage, as defined below, were consecutively recruited from those seen between July 2004 and February 2007 (inclusive) in outpatient clinics at Nihon University School of Medicine, The University of Tokyo Graduate School of Medicine, Kobe University Graduate School of Medicine, Kumamoto University Graduate School of Medicine, Saga University Graduate School of Medicine, and the Yoshikawa Eye Clinic of the Tokyo Post and Telecommunication Hospital. All patients met the following inclusion criteria: (1) demonstration of glaucoma as the only disease leading to VF damage and/or VA impairment; (2) familiarity with the VF examination, using an HFA with HFA 24-2, and production of at least two reproducible VF test results with this program in each eye, prior to enrolment in this study; (3) exhibition of a reproducible mean deviation (MD) of ≤−20 decibel (dB) in either eye, using that program; (4) BCVA of ≥20/40 (0.5) in both eyes; (5) absence of any clinically significant cataracts that might influence VF examinations during a 5-year follow-up period; and (6) demonstration of acceptable intraocular pressure (IOP) control at the time of enrolment in this study. If it was thought that the patient’s IOP was not well-controlled on the maximum tolerable dose of medication, and additional therapeutic interventions were not feasible either because of the patient’s physical condition or the patient’s wishes, then the patient could still be enrolled if all other inclusion criteria were met.

After informed consent was obtained, enrolled patients underwent a routine ocular examination that included BCVA testing, slit-lamp biomicroscopy, Goldmann applanation tonometry, dilated fundus examination and VF examination using both HFA 24-2 and HFA 10-2. All patients were required to demonstrate reliable VF measurements, with a fixation loss rate of <20%, a false-positive ratio of <15% and a false-negative ratio of <33%. After enrolment, participants received routine ophthalmological examinations, including IOP measurements, every 2–3 months, VF tests with HFA 10-2 and HFA 24-2 every 6 and 12 months, respectively, and fundus photographs every 12 months.

### Binocular IVFs

Monocular HFA 10-2 results from both eyes, obtained with a 5-minute rest interval between eyes, were combined to construct a 10-2 IVF, using the best location model^8^, and to record the best TD values from each of two corresponding locations in the right and left eyes. Nelson-Quigg *et al*. reported that either model (best location or binocular summation) provided good predictions of binocular VF sensitivity, with no significant difference in performance^8^. Thus, we chose the best location model for the current study because of its simplicity. A 24-2 IVF was also constructed in the same manner, using HFA 24-2 test results that were obtained within 3 months of VRQoL assessment.

### Assessment of VRQoL

During follow-up, the VRQoL of each participant was assessed using the questionnaire previously proposed by Sumi *et al*.^10,11^. An interview regarding the patient’s perception of visual disability was performed by a technician who was not involved in either clinical examinations or glaucoma treatments. The questionnaire contains 30 questions addressing 7 areas, including the legibility of letters (labelled as “letters”) and sentences, walking, using public transportation (labelled as “going out”), dining, dressing, and additional activities (labelled as “miscellaneous”) (Appendix)^10^. All tasks were scored on a three-category difficulty scale; 0 = greatly disabled, 1 = slightly disabled, 2 = not disabled. Raw response category scores for each question of each task were combined by simple addition to obtain a task raw score (patients’ Sumi’s questionnaire score). Total raw scores were obtained by simple addition of the task raw score for each patient (patient raw score). The relative difficulty of each task was assessed using Rasch analysis^11^, and Rasch analysis-derived easiness parameters (RADEPs) were calculated for each question of each task, as previously reported for Sumi’s questionnaire^11^. Subsequently, the RADPAI for each of the seven tasks and for all tasks combined, were calculated from the task raw scores and patient raw score. Therefore, the RADEP represents the ease with which each item can be completed, and the RADPAI reflects the overall visual ability in daily life for each subject.

The IVF TD values from each of two 10-2 IVF assessments, obtained approximately 3 months before and 3 months after the VRQoL assessment, were averaged to construct the 10-2 IVF that was compared with the results of the VRQoL assessment. TD values at each of two 24-2 IVF assessments, obtained within 3 months of the VRQoL assessment as above, were used to construct the 24-2 IVF for comparison with the results of the VRQoL assessment.

### Analyses of relationships between 10-2 and 24-2 IVFs and VRQoL

The 10-2 IVF was divided into eight subfields within 5° of the point of fixation as follows: inner/outer upper right quadrant, inner/outer upper left quadrant, inner/outer lower left quadrant, and inner/outer lower right quadrant. The 24-2 IVF was divided into upper and lower hemifields. Then, factors that significantly contributed to RADPAI for each task were identified, from among: age, BCVA in each eye, and the mTD of each eight-quadrant subfield, using the 10-2 IVF and 24-2 IVF as explanatory variables (Figs 1, 2). Because significant inter-correlation was expected among the BCVA and the mTDs of each quadrant subfield of the 10-2 IVF, and the upper/lower hemifields of the 24-2 IVF, we used the Random Forest learning algorithm^21^ to adjust for inter-correlations among variables. This algorithm, proposed by Breiman^21^, is used for classification, regression and clustering, and consists of many decision trees, where each tree is developed by bootstrapping of data and predictor variables, and results in a classification based on the consensus of all decision trees^34–37^. In the Random Forest, the following procedures are performed.

1. Feed job (out of bag) cases to each tree and perform prediction.
2. Randomly permute a specific variable (e.g., the mTD value in each region) on job cases and feed the data with permuted variables into the Random Forests model.
3. Calculate the difference in prediction accuracy (which corresponds to residual sum of squares in a simple regression analysis) between steps 1 and 2.
4. Average the decrease in accuracy over all trees in the random forests model. If a variable has no influence on prediction accuracy, then that variable is considered insignificant, and vice versa. This permutation process voids the effect of the variable on the VRQoL scores.
5. Regarding p-values, an empirical distribution is calculated in the aforementioned way, and probability under the null hypothesis is calculated based on the empirical distribution.

The Random Forest has been used in many research studies, and has been proven to be more useful than other machine-learning methods^38,39^; this algorithm allows for inter-correlation of multiple explanatory variables. Therefore, it is especially useful in analysing data that include VF test results, such as those in the current 10-2 IVF quadrant subfields and overall 24-2 IVF, which should inter-correlate with each other^17,25,26^.

We used the statistical program language R (version 2.15.0; The R Foundation for Statistical Computing, Vienna, Austria) and the Random Forest package (version 4.6-6, https://www.stat.berkeley.edu/~breiman/RandomForests/) to perform all statistical analyses.

## Supplementary information

Sumi’s vision-related quality of life (VRQoL) questionnaire and author list
