# Supplementary material for: Relationship between Vision-Related Quality of Life and Central 10° of the Binocular Integrated Visual Field in Advanced Glaucoma
Source: Sci Rep. 2019 Oct 18;9:14990. doi: 10.1038/s41598-019-50677-0 (PMC6802178; doi:10.1038/s41598-019-50677-0)
Supplement: Supplementary file 1 — Sumi’s vision-related quality of life (VRQoL) questionnaire and author list [file 41598_2019_50677_MOESM1_ESM.pdf]

Appendix.

Sumi's vision-related quality of life (VRQoL) questionnaire

Response Category Score		0	1	2
Legibility of letters: letters				
1	Can you read the headline of a newspaper?	No	With difficulty	Yes
2	Can you read small print in a newspaper?	No	With difficulty	Yes
3	Can you read words in a dictionary?	No	With difficulty	Yes
4	Can you see the numbers in a telephone director?	No	With difficulty	Yes
5	Can you make out a fare table for trains and subway?	No	With difficulty	Yes
Sentences				
6	Do you have difficulty reading and writing?	Frequently	Occasionally	No
7	When you write sentences in vertical lines, does it lean to either direction?	Frequently	Occasionally	No
8	When you read, can you find the next line easily?	No	With difficulty	Yes
Walking				
9	Do you have difficulty because of your visual problem?	Frequently	Occasionally	No
10	Can you take a walk by yourself?	No	With difficulty	Yes
11	Do you misjudge traffic signals?	Frequently	Occasionally	No
12	Do you bump into people or objects while walking?	Frequently	Occasionally	No
13	Do you stumble on the stairs?	Frequently	Occasionally	No
14	Do you fail to notice changes in the ground?	Frequently	Occasionally	No
15	Do you fail to recognize your friends until they talk to you?	Frequently	Occasionally	No
16	Do you fail to see people or cars approaching you from the side?	Frequently	Occasionally	No
Going Out				
17	Do you have difficulty going out because of your visual problem?	Frequently	Occasionally	No
18	Do you need somebody to accompany you to go to a new place?	Yes	Preferably	No
19	Can you get a cab by yourself?	No	With difficulty	Yes
20	Do you have traveling by train?	Frequently	Occasionally	No
21	Do you feel uneasy going out at night because of your visual problem?	Frequently	Occasionally	No
Dining				
22	Do you have difficulty dining because of your visual problem?	Frequently	Occasionally	No
23	Do you drop food while dining because of your visual problem?	Frequently	Occasionally	No
24	Do you spill tea while pouring into a cup?	Frequently	Occasionally	No
25	Do you have difficulty using chopsticks?	Frequently	Occasionally	No
Dressing				
26	Do you ever button up clothing in the wrong order?	Frequently	Occasionally	No
27	Can you see your face clearly in the mirror?	No	With difficulty	Yes
Miscellaneous				
28	Can you recognize people's faces on TV?	No	With difficulty	Yes
29	Do you have difficulty finding objects dropped on the floor?	Frequently	Occasionally	No
30	Do you have difficulty dialling the telephone?	Frequently	Occasionally	No

Title: Relationship between Vision-Related Quality of Life and Central 10° of the Binocular Integrated Visual Field in Advanced Glaucoma

(18/20 words)

Short Title: Central 10° visual field and visual disability

Authors: *Yoshio Yamazaki*[1], *Kenji Sugisaki*[2,3], *Makoto Araie*[3,4], *Hiroshi Murata*[3], *Akiyasu Kanamori*[5], *Toshihiro Inoue*[6], *Shinichiro Ishikawa*[7], *Keiji Yoshikawa*[8], *Hidetaka Maeda*[5], *Yuko Yamada*[5], *Akira Negi*[5], *Masaru Inatani*[6], *Hidenobu Tanihara*[6], *Satoshi Okinami*[7], *Kenji Mizuki*[9], *Koichi Mishima*[3,10], *Kenichi Uchida*[3,10] *and Shun Matsumoto*[,10]

Authors' Institutions:
[1]Department of Ophthalmology, Tokai University Tokyo Hospital, Tokyo, Japan
[2]Department of Ophthalmology, International University of Health and Welfare, Mita Hospital, Tokyo, Japan
[3]Department of Ophthalmology, University of Tokyo Graduate School of Medicine, Tokyo, Japan
[4]Department of Ophthalmology, Kanto Central Hospital of the Mutual Aid Association of Public School Teachers, Tokyo, Japan
[5]Department of Surgery, Division of Ophthalmology, Kobe University Graduate School of Medicine, Kobe, Japan
[6]Department of Ophthalmology, University of Kumamoto Graduate School of Medicine, Kumamoto, Japan
[7]Department of Ophthalmology, University of Saga Graduate School of Medicine, Saga, Japan
[8]Yoshikawa Eye Clinic, Machida, Japan
[9]Department of Ophthalmology, Nihon University School of Medicine, Tokyo, Japan
[10]Department of Ophthalmology, Tokyo Post and Telecommunication Hospital, Tokyo, Japan

Corresponding author:
Yoshio Yamazaki, M.D., Ph.D.
Department of Ophthalmology, Tokai University Tokyo Hospital, 1-2-5, Yoyogi, Shibuya-ku, Tokyo 151-0053, Japan
Tel.: +81-3-3370-2321, Fax: +81-3-5354-5366,
E-mail: yamazaki.yoshio@tokai.ac.jp
